# Lightweight, Strong and Stiff Lattice Structures Inspired by Solid Solution Strengthening

**DOI:** 10.3390/ma18091984

**Published:** 2025-04-27

**Authors:** Peijie Xiao, Shiwei Xu, Longbao Chen, Zhisheng Ruan, Zhuoran Zeng, Zhi Xiao, Jianyu Li

**Affiliations:** 1 State Key Laboratory of Advanced Design and Manufacturing for Vehicle Body, College of Mechanical and Vehicle Engineering, Hunan University, Changsha 410082, China; xiaopeijie@hnu.edu.cn (P.X.); xushiwei@hnu.edu.cn (S.X.); chenlb2022@hnu.edu.cn (L.C.); rzs521521@hnu.edu.cn (Z.R.); zeng.zhuoran@hnu.edu.cn (Z.Z.); 2 Suzhou Research Institute of Hunan University, Suzhou 215131, China

**Keywords:** lattice structures, solid solution strengthening, sosoloid structure, lightweight and high-strength, theoretical limit

## Abstract

In engineering design, introducing lattice structures offers a cost-effective method for reducing weight while enhancing load-bearing efficiency, compared to merely enhancing the material strength of a solid component. Among the various lattice structure configurations developed thus far, the strength and stiffness of these structures remain significantly below their theoretical limits. This study demonstrates that the theoretical limits of strength and stiffness in lattice structures can be achieved by mimicking the solid solution strengthening mechanism in materials science. This innovative structure achieves the highest load-bearing efficiency to date and is applicable to lattice structures of any geometric configuration. The introduction of the sosoloid structure, a lattice structure with struts reinforced along the loading direction, increases the theoretical limits of lattice strength and stiffness by 20% and 27.5%, respectively, compared to traditional uniform lattice structures. The most effective enhancement is observed when sosoloid structures exhibit the highest material utilization rate and optimal spatial layout. These findings offer a general approach to achieving high load-bearing structures and have broad application prospects in lightweight and high-strength structures, such as human bone design and energy absorption.

## 1. Introduction

Lattice structure, a network composed of a series of elements (struts, panels or surface feature unit) by periodic combination, has the advantages of low density, excellent specific performance (such as high specific stiffness and strength, etc.) and large surface area [1,2,3,4,5,6,7,8,9]. For example, lattice structures of the same strength can reduce weight by more than 70 percent compared with traditional solid materials. Therefore, it has a significant potential for application in aerospace, weapons, nuclear industry, biomedicine, automotive and infrastructure industries for high load bearing, energy absorption and heat transfer efficiency on the premise of lightweight [10,11,12,13,14,15,16]. Classical lattice structures, such as a simple cubic (SC), a body-centered cubic (BCC), a face-centered cubic (FCC) and auxetic structures [17,18,19,20,21,22,23], have been extensively studied due to the number and spatial arrangement of their nodes and struts can be flexibly adjusted to obtain the desired mechanical properties. By changing the materials and the geometric configuration, a series of new lattice structures (e.g., Kelvin, Octet truss, DHEX, IW, etc.) have been developed to achieve higher load-bearing efficiency, higher specific stiffness and strength [24,25,26,27,28,29]. However, because of the lack of an efficient and universal design method, the improvement in strength and stiffness is very limited. The relative strength (*δ/δ_s_*) and relative stiffness (*E/E_s_*) are far below the value of relative density (*ρ/ρ_s_*), where *ρ*, *σ* and *E* are density, yield strength and Young’s modulus of the lattice structure, respectively, and *ρ_s_*, *δ_s_*, *E_s_* are the corresponding values for solid bulk material [30,31]. Their relationships can be expressed by the equations of *δ/δ_s_~*(*ρ/ρ_s_*)*^n^* and *E/E_s_~*(*ρ/ρ_s_*)*^n^*, whereas exponent coefficient *n* ≥ 1. The exponent coefficient *n* value of 1 means that the theoretical limit of load-bearing efficiency is achieved. To date, the *n* values of strength and stiffness of classical lattice structures only reach 1.35 and 1.42, respectively.

Recently, some studies have demonstrated that modifying the fillet radius significantly enhances the mechanical performance of lattice structures [32,33]. This design method is important and valuable for current research because it rapidly and accurately improves the mechanical response of the lattice structures. In addition, a new method to increase the theoretical limit of lattice structures has emerged and become popular by exploiting the connection between micro and macro-structural scales in the cross field of materials science and mechanical engineering [34], namely the lattice structure, which has the same materials and geometric configurations is composed of meta-grains (domains of the same lattice orientation) [35,36]. By mimicking the crystalline microstructures and integrating metallurgical hardening principles (grain-size effect, precipitation and multiphase hardening), the strength and damage-resistance of the lattice structure are significantly improved, but the improvement is highly dependent on the number of meta-grains. This method can decrease the *n* value from 1.35 to 1.19 at maximum, but this is still much deviated from the theoretical limit *n* value of 1.

In this study, we developed a universal design method inspired by solid solution strengthening in materials science, which has further enhanced the load-bearing efficiency of lattice structures close to the theoretical limit. Solid solution strengthening is a well-established mechanism in which the strength and hardness of alloyed metals are improved due to local lattice distortion caused by the replacement of solvent atoms by solute atoms. In the microscopic crystal structures, this phenomenon can be represented by the strengthening of the corresponding atomic bonds. We integrate the principle of solid solution strengthening into the design of lattice structures. When designing the macroscopic lattice structures, it is reflected that the length of the struts remains unchanged, but their cross-sectional area becomes larger. Deploying this design principle, we obtained the highest load-bearing efficiency of lattice structures so far, with the *n* values of strength and stiffness reaching 1.08 and 1.03, respectively. The novel structure can be applied to the lattice structures of any material and geometry configuration. The purpose of this paper is to report the remarkable phenomenon and design principle.

## 2. Experimental Procedure

### 2.1. Lattice Structure Design and Fabrication

**Figure 1:**
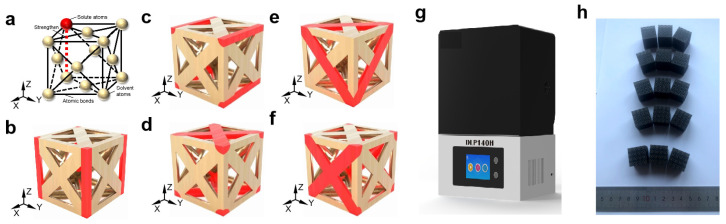
The process of sample design and preparation: (**a**) schematic diagram of sosoloid cell design inspired by the solid solution strengthening; (**b**) [001]-strengthened sosoloid; (**c**) sosoloid A: [100]-strengthened; (**d**) sosoloid C: [-110]-[110]-strengthened; (**e**) sosoloid B: [-110]-[0-11]-[-101]-strengthened; (**f**) sosoloid D: [011]-[0-11]-strengthened; (**g**) the DLP140H 3D printer (ZRapid Tech); (**h**) five FCC lattice structures.

In our design, the strength and stiffness of the structures are predominantly determined by the macroscopic lattice struts, similar to the fact that the strength and stiffness of the material are determined by the atomic bonding in the crystal lattice, as shown in Figure 1a. When there is no solid solution strengthening, all struts have the same diameter. As shown in Figure 1b, when solid solution strengthening is applied, struts along the loading direction will have a larger diameter to mimic the solid solute strengthening along the [001] crystal direction. When the struts are strengthened along the loading direction ([001] in this context, referred to as [001]-strengthened sosoloid), the diameter of the red struts along [001] is greater than that of the other struts. The base struts are strengthened by a solid solution, and the non-base struts are not strengthened by a solid solution. Figure 1c–f show the scheme of solid solute strengthening in other crystal directions.

In this paper, a 4 × 4 × 4 lattice structure based on FCC configuration is designed for sample printing and preparation. The cell size of the lattice structure is 5 mm × 5 mm × 5 mm. The overall size of the lattice structure is formed by arraying a single cell, and its size is 20 mm × 20 mm × 20 mm. The diameter of the base struts was set at 1 mm, while that of the non-base struts was 1.5 mm. Based on the DLP140H 3D printer (ZRapid Tech, Suzhou, China), we respectively used Rigid 10 material to prepare dog-bone tensile and cylindrical compression specimens to characterize the mechanical properties of the material according to ASTM D638 and ASTM D695 standards. Subsequently, we used Rigid 10 material to fabricate the 4 × 4 × 4 FCC lattice structures. The printing process parameters used are as follows: the printing layer thickness is 0.05 mm, the laser power is 100 mW, the substrate exposure time is 10 s, the support exposure time is 3.3 s, and the physical exposure time is 1.5 s. Figure 1h shows the five FCC lattice structure samples of the solid solution strengthening in this paper.

The other lattice structures (FCC, BCC) discussed in this paper are arranged in a 4 × 4 × 4 configuration along the X, Y and Z directions. The cell size ranges from 1 mm to 15 mm, and the overall size ranges from 4 mm to 60 mm. All the lattice CAD models presented in this paper are designed using UG NX 12.0 software. The traditional uniform lattice structure and the lattice structure in this paper are composed of the sosoloid and are formed by arraying a single cell.

### 2.2. Mechanical Tests and Analyses

We performed tensile and compressive tests using a displacement-controlled INSTRON 1342 electronic universal testing machine (INSTRON Corporation, Wycombe, UK, maximum load 10 kN) at room temperature to test the basic mechanical properties of the material and all lattice structures. Here, the strain rate is 10^−3^ s^−1^. The loading direction of the displacement is always parallel to the build direction (Z direction) of the print. The engineering stress is calculated by dividing the recorded force by the cross-sectional area perpendicular to the Z direction (the area of the rectangle formed by the outer dimensions). Engineering strain is calculated by dividing the change in length along the Z direction by the initial length. We use the linear portion of the stress–strain curve, offset by 0.2% strain, as it is parallel, and its intersection with the stress–strain curve determines the yield stress [35]. We use the most linear part of the stress–strain curve to determine Young’s modulus by fitting a linear function.

### 2.3. Finite Element Analysis (FEA)

**Figure 2:**
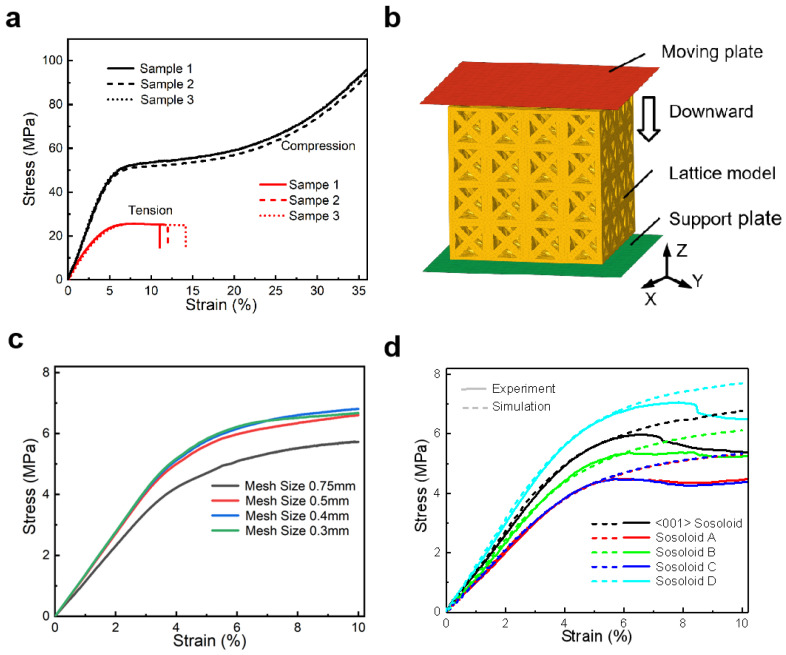
Experimental and numerical results: (**a**) the stress–strain curve of tension and compression of Rigid 10 material; (**b**) simulation model; (**c**) the mesh convergence of the model; (**d**) the comparison of experimental and simulated stress–strain curves.

The lattice structures are divided into solid element meshes (*SECTION_SOLID), and the material card is *MAT_PLASTICITY_COMPRESSION_TNSION. The mechanical properties data for simulating the asymmetric behavior of tension and compression of isotropic elastic-plastic materials are shown in Figure 2a. When the actual mechanical property data are input, it is necessary to convert the engineering stress–strain obtained from the test into the true stress–strain. Among them, the material density is 1.2 g/cm^3^, the tensile and compressive elastic modulus are 730 GPa and 1030 GPa, respectively, and the Poisson’s ratio is 0.37. Both the compression (moving) and support panel are rigid planes. The element type is a shell element, the material card is *MAT_RIGID, the material density is 7.8 g/cm^3^, the elastic modulus is 210 GPa, and the Poisson’s ratio is 0.3. The contact between the rigid plane and the lattice structures is defined as surface-to-surface contact, and the lattice structure itself is an automatic general contact. The type of contact algorithm used for surface-to-surface interactions is the Penalty Method. Both their static and dynamic friction coefficients are 0.2. The damping parameters of the model use the default viscosity parameters, with Q_1_ set to 1.5 and Q_2_ set to 0.06. The support panel constrains six degrees of freedom, and the compression panel constrains the remaining five degrees of freedom, except for the Z-direction movement. Then, we solve it on the LS_DYNA solver.

To validate the quasi-static compression simulation, we conducted uniaxial compression experiments on [001]-strengthened sosoloid lattice structures under identical conditions. The hourglass energy is less than 5% of the internal energy, and the calculation results are stable and reliable. As shown in Figure 2c, when the mesh size of the model is half the diameter of the base struts, it has the best computational efficiency and accuracy. Therefore, the mesh division rules in this paper are unified to half of the minimum strut diameter. Prior to the onset of buckling, we found that the numerical and experimental results were closely aligned. Besides the [001]-strengthened lattice, the quasi-static compression of [100]-strengthened, [-110]-[0-11]-[-101]-strengthened, [-110]-[110]-strengthened and [011]-[0-11]-strengthened lattices were also simulated and subsequently validated through testing, as shown in Figure 2d. The results demonstrated a strong correlation between the simulations and experiments, confirming that our model can effectively simulate the elastic behavior of all lattice structures.

## 3. Results and Discussion

### 3.1. Strength and Stiffness of Lattice Structures Along the Loading Direction

**Figure 3:**
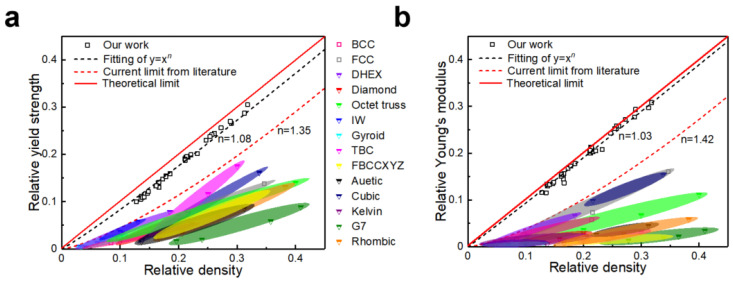
The relationship between (**a**) relative yield strength and (**b**) relative Young’s modulus as functions of the relative density of lattice structures that are strengthened along the loading direction [11,21,22,23,28,29,37,38,39,40,41,42].

When the struts aligned with the loading direction ([001] struts) are strengthened, as shown in Figure 1a,b, the [001]-strengthened sosoloid lattice structure demonstrates a significant improvement in load-bearing capacity. Figure 3a,b provide a schematic illustration of solid solution strengthening and a sosoloid cell, highlighting its strength and stiffness. It presents the relationship between relative yield strength and relative Young’s modulus as functions of the relative density of lattice structures that are strengthened along the loading direction. The values were obtained through the simulation of quasi-static compression tests using LS_DYNA R11. Under the optimum design, the *n*-value of strength and Young’s modulus decrease from 1.35 to 1.08 and from 1.42 to 1.03, respectively (Figure 3a,b). By comparing with the existing struts-based lattice structures [11,21,22,23,28,29,37,38,39,40,41,42], it is demonstrated that the stiffness and strength of the structure have been greatly improved, which makes it approach the theoretical limit to the maximum extent.

### 3.2. Effect of Lattice Geometry and Configuration on Strength and Stiffness

**Figure 4:**
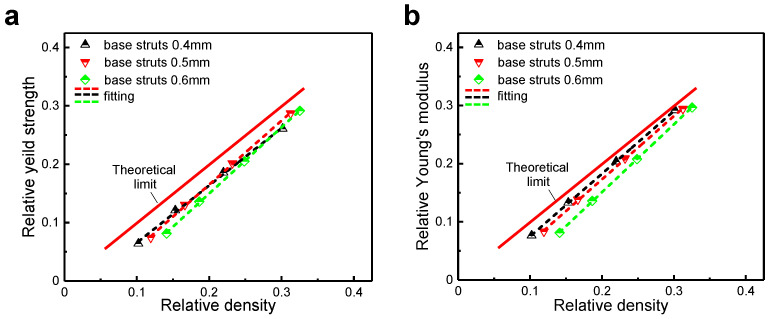
The influence of lattice geometry on its strength and stiffness: (**a**) yield strength under different base strut diameters; (**b**) Young’s modulus under different base strut diameters.

For the lattice structure composed of the [001]-strengthened sosoloid, its geometry and configuration have an effect on whether the strength and stiffness can reach the theoretical limit value. This will be related to the stability of the lattice structure. For lattice geometric, when the diameter of the base struts is reduced from 0.5 mm to 0.4 mm, the lattice structures begin to be unstable, which is manifested by Young’s modulus remaining close to the theoretical limit, while its yield strength decreases significantly (Figure 4) (taking a three-dimensional lattice structure composed of the [001]-strengthened sosoloid with a macroscopic global size of 20 mm × 20 mm × 20 mm and the sosoloid length of 5 mm as an example). The lower the base strut diameter, the more unstable the lattice structures in the elastic stage. It is equivalent to the lattice structures without base struts losing their bearing capacity under a small external force.

**Figure 5:**
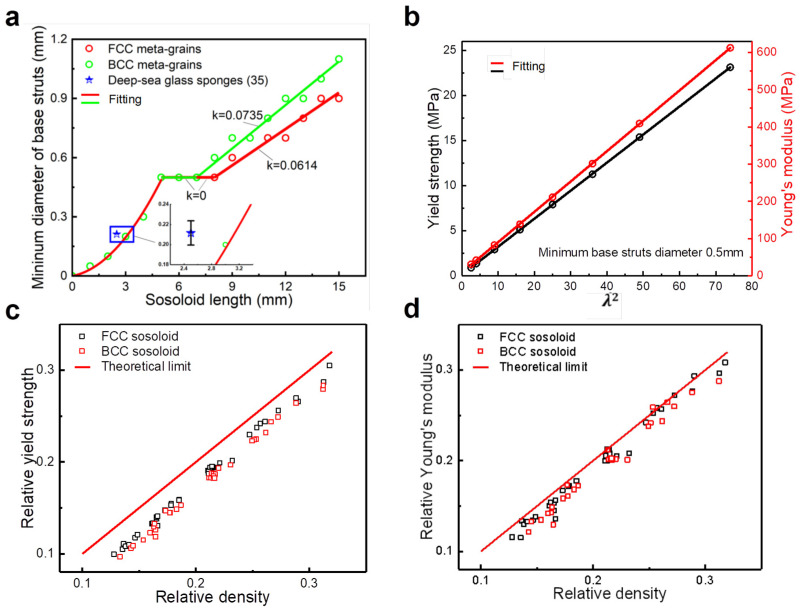
Effects of geometry and configuration of [001]-strengthened sosoloid on mechanical properties and numerical results: (**a**) the relationship between the minimum base strut diameter and sosoloid length; (**b**) the mechanical properties of sosoloids with different grain sizes and their quadratic relationship; (**c**,**d**) the comparison of the relative yield strength and relative Young’s modulus of FCC and BCC sosoloids.

For the limit boundary of 5 mm sosoloid length, the minimum base strut diameter can be approximately 0.5 mm. Meanwhile, for lattices of other sosoloid length dimensions, we find that the minimum base strut diameter and sosoloid lengths follow a quadratic function for sosoloid lengths below 5 mm. For sosoloid lengths above 5 mm, the minimum diameter of the base struts has a two-segment linear relationship with sosoloid lengths (Figure 5a). Under different sosoloid lengths and minimum diameters of the base struts, the ratio (*λ*) between the [001] struts diameter (*d*) and minimum base struts diameter (*d*_m_) is greater than a certain value when the lattice reaches the theoretical limit value. Here, we can describe the ratio (*λ*) by introducing evenness, namely *λ* = *d*_m_/*d*. It is worth noting that there is a quadratic relationship (*σ_y_* = *σ*_0_ + *kλ*^2^) (Figure 5b). By fitting the simulation data, we can obtain *σ*_0_ = 0.08 MPa, *k* = 0.31 MPa of determination *R*^2^ = 99.99%. In addition, we found that Young’s modulus also has a similar relationship (*E_y_* = *E*_0_ + *kλ*^2^), which is *E*_0_ = 6.77 MPa, *k* = 8.2 MPa, coefficient of determination *R*^2^ = 99.98%.

For lattice configurations (FCC and BCC), the relationship between the minimum base struts diameter and sosoloid lengths also is a quadratic function first and then a two-segment linear relationship. Therefore, the sosoloid geometric configuration can affect the two-segment linear relationship, which is manifested as the difference between the platform length of the first horizontal line relationship and the slope size of the two-segment linear relationship. This is caused by the different spatial arrangement of BCC and FCC sosoloid, which is similar to that the face-centered cubic lattice is denser than the body-centered cubic lattice due to the difference in coordination number and density at the crystal structures level. As a consequence, the face-centered cubic structures have better spatial stability than the body-centered cubic structures. It is worth noting that when the sosoloid length is less than 5 mm, the geometric configuration has no effect on the minimum base strut diameter in Figure 4a. But it does matter, in fact, just a little. Moreover, the smaller the sosoloid length is, the less the influence of the geometric configuration on the minimum base strut diameter. But the most remarkable thing is that the results of mechanical properties analysis for the lattice structures composed of FCC and BCC sosoloid have a consistent theoretical limit value (Figure 5c,d).

### 3.3. Mechanism of [001]-Strengthened Sosoloid Achieving Limits of Theoretical Properties

**Figure 6:**
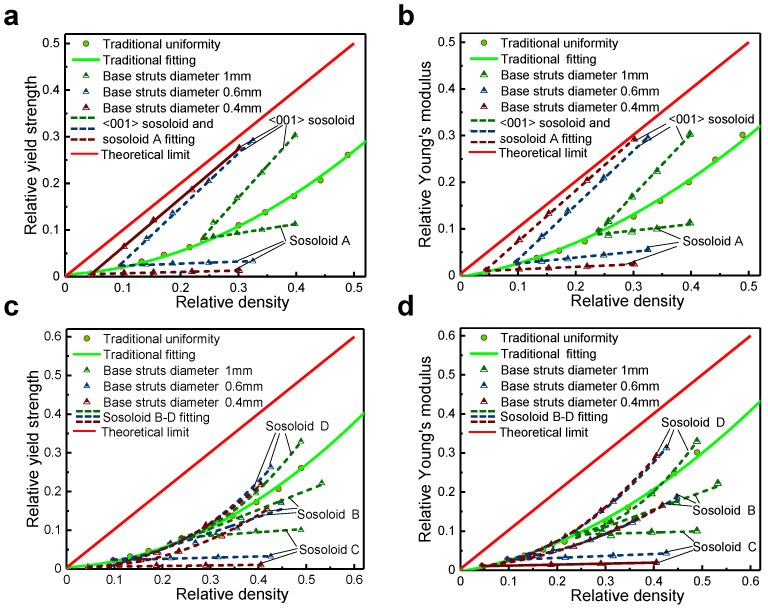
The relationship between relative yield strength, relative Young’s modulus and relative density for lattice structures composed of [001]-strengthened and other sosoloids: (**a**,**b**) [001]-strengthened sosoloid and sosoloid A; (**c**,**d**) sosoloids B through D.

The lattice structure composed of [001]-strengthened sosoloid with different parameters will have the highest specific stiffness and specific yield strength within a reasonable boundary. In fact, there are also a series of non-[001]-strengthened sosoloid lattice structures that also are inspired by solid solution strengthening (Figure 1c,f). However, their mechanical properties are not up to the theoretical limit. Only the [001]-strengthened sosoloid lattice structure can be achieved. Here, the other four sosoloid lattice structures are formed by combining the crystal orientations. They are respectively [100], [-110]-[0-11]-[-101], [-110]-[110], [011]-[0-11]-strengthened sosoloid lattice structures (Figure 1c–f). As shown in Figure 1g, we can find that the initial elastic response characteristics of [001]-strengthened sosoloid and sosoloid A-D have large differences, which indicates that sosoloid strengthening design schemes have a greater impact on the initial overall structural stiffness and yield strength of the lattice structures. Combined with Figure 6a–d, it was found that the material distributed along the axial loading direction has a greater impact on the structural stiffness and yield strength, and its material utilization rate is the highest. [001]-strengthened sosoloid not only broadens the bearing capacity of structures but also approaches the maximum theoretical limit.

**Figure 7:**
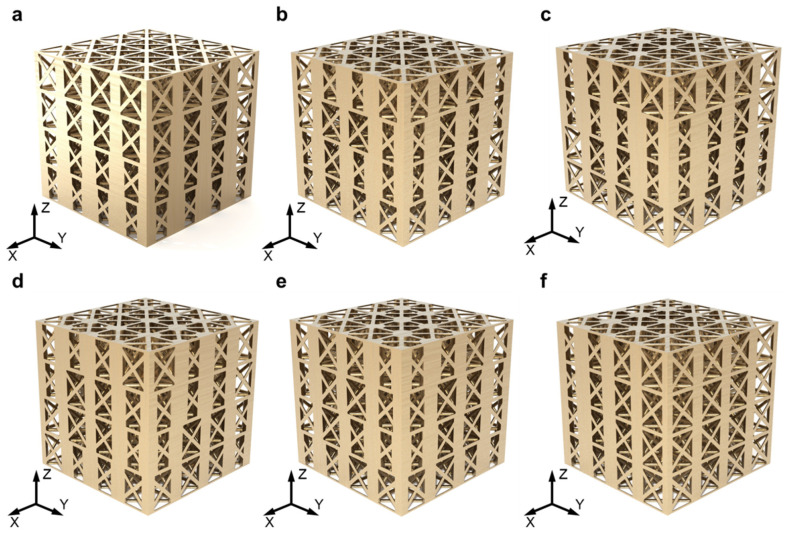
The 3D models of a series of lattice structures composed of [001]-strengthened sosoloid are described as follows: (**a**) gradient lattice structures with [001]-strengthened sosoloid (S2); (**b**) random point-enhanced lattice structures (S3); (**c**) random line-enhanced lattice structures (S4); (**d**) random surface-enhanced lattice structures (S5); (**e**) random body-enhanced lattice structures (S6); (**f**) directed-enhanced lattice structures (S7).

**Figure 8:**
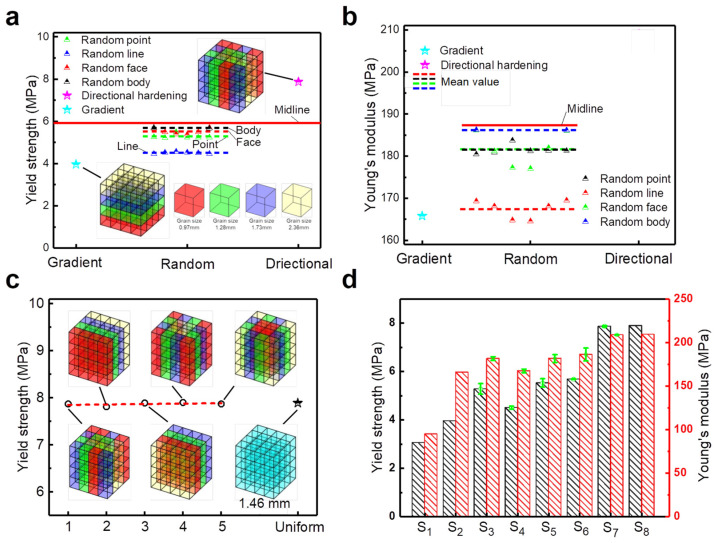
Mechanical properties of various lattice structures composed of [001]-strengthened sosoloid: (**a**,**b**) yield strength and Young’s modulus of the lattice structures of gradient, randomly enhanced (point, line, surface, and volume) and directionally enhanced; (**c**) yield strength of directionally enhanced lattice structures with different arrangements under equal mass; (**d**) mechanical properties of various lattice structures (S1–S8).

On the other hand, the lattice structure composed of [001]-strengthened sosoloid still possesses the optimal spatial layout because of its uniform size and directional arrangement and achieves the highest mechanical properties compared to other types of lattice structures under the same mass. We can stack the [001]-strengthened sosoloid of the same and different grain sizes in an orderly and regular manner in the three-dimensional filling space to become various lattice structures at the same mass (Figure 7). Among them, the gradient lattice structures with the four grain sizes (*d* = 0.97 mm, 1.28 mm, 1.73 mm and 2.36 mm) (Figure 8a) were compared with the uniform lattice structures without sosoloid (S_1_) under the equal mass conditions (with all strut diameters evenly distributed at 0.99 mm). The yield strength and Young’s modulus of the gradient lattice structures with the sosoloid increased by 29.41% and 74.76%, respectively (S_1_ and S_2_ of Figure 8d). In fact, the gradient lattice structures usually show very poor mechanical properties and always begin to buckle and fail from the minimum strut diameter and gradually increase [42]. It is attributed to the huge enhancement effect brought by sosoloid to offset the strength-weakening effect of the gradient lattice structures under the premise of efficient utilization of materials. In addition, when sosoloids of the same grain size are axially aggregated (directionally enhancement lattice structures, S_7_), the axial hardening effect just completely cancels the strength weakening effect of the gradient lattice structures, thus achieving a [001]-strengthened sosoloid lattice structures (S_8_) with equal mass and properties (Figure 8c). When sosoloids of the same grain size are dispersed and arranged, the axial hardening effect and radial weakening effect of the randomly enhancement lattice structures become a competing mechanism. Also, their performance is lower than the average performance (midline) of the horizontal gradient lattice structures and directionally enhancement lattice structures with [001]-strengthened sosoloid (Figure 8a,b).

## 4. Conclusions

This paper proposes novel lightweight, strong and stiff lattice structures composed of a [001]-strengthened sosoloid, which exhibit excellent properties by simulating the solid solution strengthening mechanism of materials. The novel structure can be applied to the lattice structures of any material and geometry configuration. The main conclusions are as follows:

(1) Compared to other lattice structures, the [001]-strengthened sosoloid lattice structure demonstrated a significant improvement in load-bearing capacity. The n values for the strength and stiffness of the [001]-strengthened sosoloid structure reach 1.08 and 1.03, respectively, approaching the theoretical maximum limit of 1 for the first time.
(2) The lattice structure composed of [001]-strengthened sosoloid can withstand higher loads and is applicable to lattice structures of various geometric configurations. The results of the mechanical property analyses for the lattice structures composed of FCC and BCC sosoloid have a consistent theoretical limit value.
(3) Sosoloids with uniform grain size can enhance the mechanical properties of lattice structures while also achieving optimal structural isotropy. This optimal isotropy maximizes the mechanical properties of lattice structures under uniaxial loading conditions. This is attributed to the [001]-strengthened sosoloid lattice structure’s high material utilization rate and optimal spatial layout.

## Data Availability

The raw/processed data required to reproduce these findings cannot be shared at this time, as the data also form part of an ongoing study.
